# Effects of Die Configuration on the Electrical Conductivity of Polypropylene Reinforced Milled Carbon Fibers: An Application on a Bipolar Plate

**DOI:** 10.3390/polym10050558

**Published:** 2018-05-22

**Authors:** Nabilah Afiqah Mohd Radzuan, Abu Bakar Sulong, Mahendra Rao Somalu, Edy Herianto Majlan, Teuku Husaini, Masli Irwan Rosli

**Affiliations:** 1 Fuel Cell Institute, Universiti Kebangsaan Malaysia, Bangi, 43600 UKM, Malaysia; abubakar@ukm.edu.my (A.B.S.); mahen@ukm.edu.my (M.R.S.); edyhm71@gmail.com (E.H.M.); t-husaini@ukm.edu.my (T.H.); 2 Department of Mechanical and Materials Engineering, Faculty of Engineering and Built Environment, Universiti Kebangsaan Malaysia, Bangi, Selangor 43600, Malaysia; 3 Department of Chemical and Process Engineering, Faculty of Engineering and Built Environment, Universiti Kebangsaan Malaysia, Bangi, Selangor 43600, Malaysia; masli@ukm.edu.my

**Keywords:** carbon fibers, electrical conductivity, extrusion, bipolar plate

## Abstract

Die configurations, filler orientations, electrical conductivity, and mechanical properties of polypropylene reinforced milled carbon fibers were studied as functions of their manufacturing processes. Series of manufacturing processes often deteriorate the material properties, hence, finding a suitable process aid is key to improving the electrical and mechanical properties of composite materials. Compared with the conventional manufacturing process, extrusion is a key process in the production of a highly conductive composite. A twin-screw extruder was used at a temperature of 230 °C and a rotational speed of 50 rpm before the compression molding process was carried out at 200 °C and 13 kPa. This research examined different die configurations, namely rod and sheet dies. The results indicated that the rod dies showed better mechanical properties and electrical conductivity with 25 MPa and 5 S/cm compared to the sheet dies. Moreover, rod dies are able to orientate to 86° and obtain longest filler length with 55 μm compared to the sheet dies. The alteration of the filler orientation in the produced material at a high shear rate further enhanced the electrical conductivity of the material.

## 1. Introduction

Pre-mixing is a method of chaotic mixing before a material undergoes manufacturing processes such as injection molding and compression molding. These processes allow the fibers to be pulled out of the bundles and oriented along the flow direction in order to construct a conductive pathway [1]. Yet, the process has not conventionally applied to the study regarding its effect as still being preliminary. Researchers have often reported that the conventional process, including injection and compression molding, is able to enhance the mechanical properties of the end product [2]. However, as the mechanical properties increase, the electrical conductivity tends to decrease. This is important, especially in producing composite materials used in bipolar plate applications for polymer electrolyte membrane fuel cell (PEMFC) applications [3]. Developing an excellent electrical conductivity while maintaining the mechanical properties of composite materials is the main challenge which has not yet been resolved. Therefore, melt mixing in a twin-screw extruder is the main alternative in the development of highly conductive polymer composite materials by controlling the fiber orientations. The components of the materials are physically pre-mixed, blended, and melted in the extruder machine [4]. Twin screw extruders are widely used in various industries including polymer, food, and material processing industries due to their ability to run multiple processes (heating, compounding, blending) continuously [5,6]. In the extrusion process, the die permits the fiber to be oriented along the extruded direction. Thus, a proper design of the die configuration is extremely important in order to produce an accurately orientated fiber in the extruded product as the die functions to shape the molten composite exiting the extruder into the desired orientations [7]. Besides, the extruded material usually swells to a size that is bigger than the diameter of the die orifice, thus making the die configuration even more crucial [8].

The orientation of the fiber in a polymer matrix can be induced into an aligned direction in several ways, including by means of the shear rate, die size, filler size, and processing technique [9,10,11]. The shear rate is able to align the fiber accordingly as the fiber tends to disperse and orientate in the extrusion direction [12,13]. Meanwhile, the geometry of the die can be altered to obtain a suitable orientation depending on the application [11]. Besides the fiber size, the fiber aspect ratio also aids in inducing the orientation of the fiber, where a shorter fiber orientates more randomly than a longer filler due to the high shear stress experienced by the latter [14]. The die shapes that are commonly used are either diverging or converging, depending on the application. In 1995, it was reported that the diverging die permits the fibers to orientate randomly over the entire thickness in the extrusion direction, thereby producing the elongation deformation that will create the desired orientation. However, the shear deformation near the die wall obstructs the fibers from orientating accordingly [15]. Unlike the diverging die, the converging die induces a compact and dense longitudinal fiber orientation throughout the die and enhances the mechanical properties and electrical performance of the material. In 1995, Hine reported that converging dies are more effective than strip dies due to their ability to align the fibers along the flow direction while producing a high degree of fiber alignment [16]. Meanwhile, the diameter of the die opening depends on the material used as different materials offer different shear rates. There are several studies that have involved different die geometries, including the use of a carbon fiber/polypropylene composite material with a die opening size of 2 mm and a circular die with a diameter of 1 mm for PP/CaCO_3_ [8,11]. This explains why both the shape and the die geometry are crucial for obtaining good electrical conductivity and mechanical properties. Besides, when attempting to understand the fundamental method for inducing orientation, a major challenge in the orientation factor is the difference between each particle [9,17]. It is suggested in this research that shear helps to induce fiber orientation within a polymer matrix and minimises the fiber breakage [18]. At higher filler loadings, it is more difficult for a polymer resin to wet the filler particles, thus leading to further complications during compounding, such as die blockage [19].

Meanwhile, instead of the mixing and manufacturing processes, material selections of conductive polymer composites (CPC) are also crucial to obtain excellent electrical conductivity and mechanical properties. CPC materials are produced by combining an insulating polymer matrix with a conductive filler, which is extensively used in various applications such as bipolar plates in fuel cells. CPC exhibits several interesting features including good thermal resistivity, good mechanical properties, and improved electrical performance [20,21]. However, CPC materials have a major difficulty in that their electrical and mechanical performances are both dependent on the volume fraction, filler dispersion, and morphology of the composites [22]. The fiber is usually orientated randomly when it is produced by compression molding [16]. The fiber dispersion is one of the most important parameters as it helps to improve the electrical conductivity of composite materials [1,23]. Studies using a glass fiber reinforced polymer showed that by aligning the filler into the desired orientation, the mechanical properties of the polymer composite can be improved [24,25]. The development of a polymer composite based on milled carbon fiber and polypropylene was fabricated. According to the literature, polypropylene is extensively used in the automobile industry and in the manufacture of domestic products such as electronic appliances, plastic storage boxes, and so on [26,27]. Meanwhile, carbon fibers are made up of at least 90 wt % carbon, which is the primary source of the mechanical strength of conductive polymer composites in terms of hardness, fracture toughness, and flexural strength [19,28].

The aim of this study is to apply a different approach by using an extrusion process incorporating the high aspect ratio filler, which results in a 90° fiber orientation. The orientated fiber will aid in developing an excellent conductive pathway and thus increase the electrical conductivity while maintaining the mechanical properties of composite materials. Despite that, the modified Fiber Contact Model (FCM) was also adapted to the study in order to predict the electrical conductivity of composite materials.

## 2. Electrical Conductivity Model

The electrical conductivity of composite materials can be predicted using the mathematical model that was commonly used by previous researchers, namely the General Effective Media (GEM) model [29,30]. This model is able to effectively predict the electrical conductivity of a polymer composite consisting of either a single filler or multiple fillers [3,31,32]. However, the latest studies have reported that the GEM model is not adequate for the prediction of electrical conductivity as the model does not consider parameters other than the volume fraction of the composite and the electrical conductivity of the filler. Therefore, the modified Fibre Contact Model (FCM) by Weber and Kamal seems to be more suitable for predicting the electrical conductivity of CPC materials as the model takes into account parameters such as the filler orientation, volume fraction, contact number of fibers, and critical filler diameter [14,30]. The angle of orientation is manually measured using the SEM image analysis. The modified FCM model is defined as Equation (1) [30]:

$$\sigma_{c}=\sigma_{m}+\left[\frac{4}{\pi }\left(\frac{d_{c}}{d}\right)\left(\frac{l}{d}\right)\left(\cos^{2}\theta \right)\left(\varphi_{overall}\sigma_{f}X\right)\right]$$

where $\sigma_{c},\sigma_{m},$ and $\sigma_{f}$ are the electrical conductivities of the composite material, matrix, and fibres, respectively; *X* is the contact number of fibres; *d* is the diameter of the fibre; *d_c_* is the diameter of the filler contact; *l* is the filler length; *φ* is the volume fraction; and *θ* is the filler orientation angle. The details of the model have been discussed elsewhere [14,30].

## 3. Materials and Method

Milled carbon fiber (MCF) with a diameter of 9 μm and an average length of 300 μm, an aspect ratio of 43, and a density of 1.75 g/cm^3^, as reported by the manufacturer, was obtained from (ShenZhenYataida High-Tech. Co., Ltd., Shenzhen, China). MCF has the ability to enhance electrical conductivity at low filler loadings, disperses evenly in a polymer matrix through the extrusion process, and has a high aspect ratio which produces high electrical conductivity [33,34]. Meanwhile, polypropylene (PP) powder grade Titan (600) with a maximum average size of 90 μm, density of 910 g/cm^3^, and melting index of 10 g/10 min at 160 °C was supplied by (Goonvean Fibers Ltd., Honiton, UK). This type of PP was chosen due to its impressive properties such as good mechanical strength, low density, electrical resistance, and greater permeability by gasses [19].

**Figure 1:**
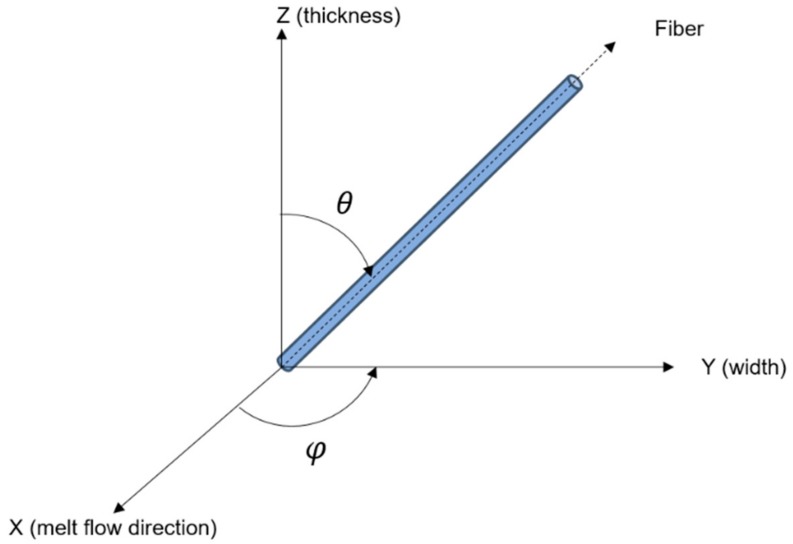
Orientation angles definition.

The compositions of 70 wt % of MCF and 30 wt % of PP were used in this study to produce highly conductive composites [23,29]. The PP and MCF composites were physically pre-mixed in a small container at room temperature using a mechanical mixer, model RM 20-IKA-WERK (Universiti Kebangsaan Malaysia, Selangor, Malaysia) at 1200 rpm for 2 to 4 min, before being melted and compounded in a Thermo Haake TSE twin screw extruder (Selangor, Malaysia). In the extrusion process, the twin screw extruder was set at a temperature of 230 °C and a rotational speed at 50 rpm for 30 min of extrusion time. Three different types of dies were used in this study, including sheet dies with thicknesses of 3 and 5 mm, and rod dies with a diameter of 5 mm. The die with a 10 mm width was used in this study with an L/D ratio of 10 [35]. These die geometries were chosen as the converging die offers a better filler orientation compared to the diverging die [16]. Recent studies show several methods used in order to measure the orientation angles, including shear light-scattering, data image processing with SEM, second-order tensor, and 2D-image analysis [36,37]. However, researchers often use the image analysis method incorporating the FESEM compared to other computational methods. Moreover, a recent study also used microstructural image analysis to study the fiber orientation in composite materials [38,39]. Thus, in this study, the orientations were observed at the fracture surface and measured manually using the Microsoft Visio based on the single fiber orientation in the Cartesian coordinate, as shown in Figure 1 [9]. The *X*-axis denotes the melt flow direction (parallel to the extrusion direction) in which the orientation angle measured is close to 90°. In contrast, if the filler orientates perpendicular to the extrusion direction, the orientation angles will be measured between 0° to 89°. Later, the extruded composite will be crushed using a crusher and compressed accordingly at 200 °C and cured at the molding pressure of 13 kPa for 15 min [26,29]. The extrusion process was used to orientate the fiber, while the compression molding process was used in producing composite materials [29,40].

Thermal degradation behavior of the milled carbon fiber reinforces polypropylene (MCF/PP) composite material was identified using thermogravimetric analysis (TGA, 851e Mettler Toledo, Quantum Skynet, Selangor, Malaysia) at a temperature ranging between 25 to 900 °C and at a heating rate of 20 °C /min in nitrogen gas conditions [23]. Meanwhile, phase characterization and theoretical density were determined using X-ray diffraction (XRD), model Bruker AXS, D8-Advance, Universiti Kebangsaan Malaysia, Selangor, Malaysia at room temperature with Cu K*α* (*λ* = 0.15406 nm) radiations. The in-plane electrical conductivity of the composite parallel to the extrusion direction was measured by the four-point probe technique using a Jandel four-point probe (Universiti Kebangsaan Malaysia, Selangor, Malaysia) and an RM3 test unit, while the through-plane conductivity was measured with a through-plane electrical conductivity tester manufactured by ZBT in Duisburg, Germany conducted at room temperature [23,26]. Besides that, the morphological structure of the composite material was observed using a Zeiss Scanning Electron Microscope (SEM, Universiti Kebangsaan Malaysia, Selangor, Malaysia) and Quanta FEI Quanta 400F (FESEM, Quantum Skynet, Selangor, Malaysia) manufactured by FEI in the USA. In order to investigate the mechanical properties of the polymer composite material, the density of the composite was measured according to the ASTM D792 standard using the Archimedes method, while the shore hardness (scale D) was measured by using a Digital Shore Hardness tester (Universiti Kebangsaan Malaysia, Malaysia) based on ASTM D2240 [23,41]. The standard tensile strength and flexural strength of the composite were measured at room temperature at a crosshead speed of 1 mm/min using an Instron 5567 Universal Testing Machine (Universiti Kebangsaan Malaysia, Malaysia) based on ASTM D3039 and ASTM D790-03, respectively [42,43]. The dimensions used for the flexural and tensile tests were 100 mm (L) × 10 mm (W) supported by a span length that was fixed at 25 mm.

## 4. Results and Discussion

### 4.1. Thermogravimetric Analysis

**Figure 2:**
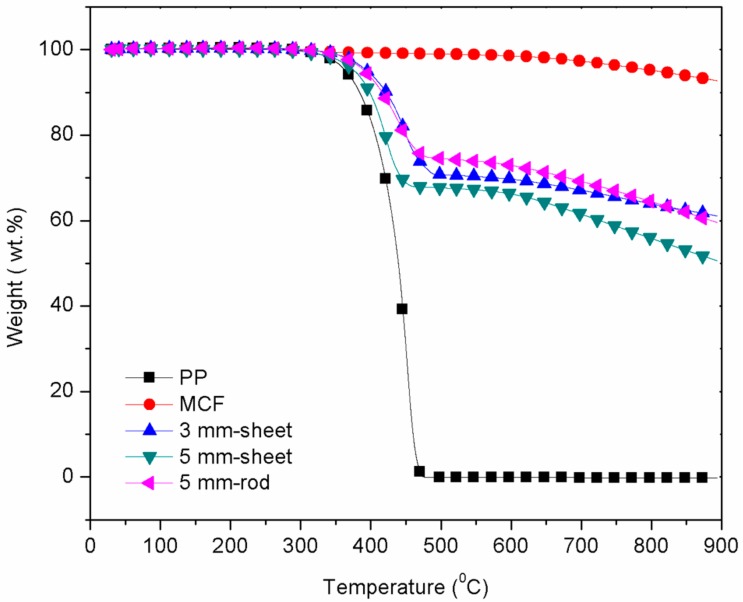
TGA curve of MCF/PP polymer composite at different die thicknesses.

Figure 2 shows the thermogravimetric analysis plot for the extruded MCF/PP polymer composite where the thermogravimetric temperature shifted at 290 °C for the pure PP and increased to approximately 310 °C for the extruded MCF/PP composite. The high thermal stability of the extruded MCF/PP composite enhanced the overall mechanical properties and allowed the material to be operated at both low and high operating temperatures [41]. The composite produced using the rod die encountered a minimum weight loss at higher temperatures compared to those produced using the sheet dies, as displayed in Figure 2. Meanwhile, for the extruded composites produced using the sheet dies, the thermogravimetric temperature shifted drastically at 292 °C as the filler was coated all over with more of the polymer matrix, thus increasing the weight loss of the composites, as shown in Figure 4a,b.

### 4.2. X-ray Diffraction Analysis

**Figure 3:**
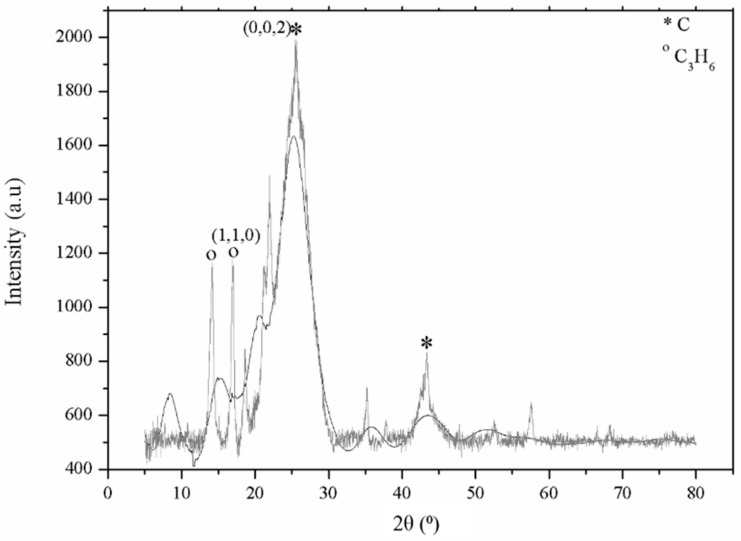
Reivtveld analysis of the MCF/PP composite.

The phase of MCF/PP composites was identified by XRD analysis, as shown in Figure 3, where the major peaks match the pure carbon (C) and polypropylene (C_3_H_6_). The highest intensity obtained for element C is observed at ~28° and ~47°, whereas the peaks at ~13° are designated to C_3_H_6_ [44]. The sharpness peaks indicated the crystal structure, which corresponded to the TGA analysis results [45]. The lattice parameter for the MCF/PP composite was 4.002 Å, which was calculated using Equations (2) and (3) [46].

*a = d √h^2^ + k^2^ + l^2^* (2)

*d* = *λ*/2 sin*θ* (3)

where *a* is the lattice parameter; *d* is the planar spacing; *h*, *k*, *l* are the Miller indices; *λ* is the X-ray wavelength (1.5406 Å); and *θ* is the peak position. Thus, the theoretical density (*ρ_TH_*) was calculated using the lattice parameter obtained using Equation (4):

(4) $$\rho_{\mathrm{TH}}=\frac{4.MW}{NA.a}$$

where *MW* is the molecular weight and *NA* is Avogadro’s number. Therefore, the calculated theoretical density obtained is 1.243 g/cm^3^, which is slightly higher than the density obtained by the experiment, as shown in Table 1. Thus, the relative density was calculated using Equation (5) [47]:

*ρ*_r_* = ρ*_s_/*ρ*_TH_ (5)

where *ρ_s_* is the experimental density and *ρ_TH_* is the theoretical density of the composite. The relative density increases as the die thickness increases, as shown in Table 1.

### 4.3. Electrical Conductivity

**Figure 4:**
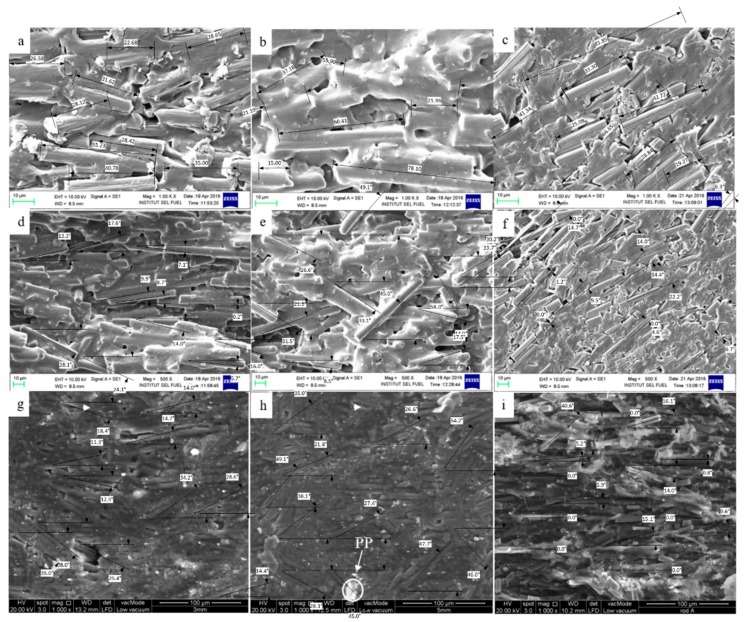
SEM images of extruded MCF/PP using 1.00 K × magnification of (**a**) 3 mm thick sheet die; (**b**) 5 mm thick sheet die; (**c**) 5 mm diameter rod die, 500 × magnifications of (**d**) 3 mm sheet die; (**e**) 5 mm sheet die and (**f**) 5 mm diameter rod die and compressed MCF/PP using 1.00 K × magnification of (**g**) 3 mm thick sheet die; (**h**) 5 mm thick sheet die; (**i**) 5 mm diameter rod die.

Figure 4 shows the fibre length and orientation angles for different types of die including the 3 mm and 5 mm sheet die and 5 mm diameter rod die. Figure 4a,b shows that the fibre experienced higher breakage compared to Figure 4c due to the high shear rate at the narrowed die orifice. The 3 mm sheet dies shown in Figure 4a have a shorter fibre length of 28 μm compared to the 5 mm sheet die shown in Figure 4b with a value of 42 μm. This phenomenon was due to the high shear rate which transferred onto the fibre, which results in higher fibre breakage. These similar phenomena are also reported when using glass fibre reinforced polypropylene composites, as fibre tends to break into a shorter size at higher rotational speeds [12]. Meanwhile, Figure 4c shows a longer fibre length of 55 μm, which is the longest compared to the sheet die. Studies using glass fibre reinforced polypropylene have suggested that the converging dies enable the fibre to orientate uniformly compared to diverging dies [16,33]. Moreover, Figure 4f clearly shows where the filler tended to orientate uniformly in the extrusion direction as the die thickness became narrower, with an average orientation angle of 83°. Meanwhile, the different observations are shown in Figure 4d,e where the fibre tends to orientate randomly in the polymer matrix using the sheet die with the thickness of 3 and 5 mm, with an average orientation angle of 77° and 68°, respectively. Studies carried out using polycarbonate with multiwalled carbon nanotubes reported that the lower orientations angles obtained were due to a low viscosity, low shear rate, and longer cooling time, which results in fibre that is able to orientate randomly [48]. The random orientation of the filler was due to the low shear stress that was transferred to the filler as the die thickness became wider [48]. Meanwhile, the MCFs tended to orientate parallel to the extrusion direction when the rod dies with a diameter of 5 mm were used, as seen in Figure 4f. Therefore, the rod die was more effective than the sheet die in aligning the filler in the extrusion direction due to fact that the uncertainties in the filler distribution in the sheet die affected the filler orientation itself [16]. In addition, the narrow sheet dies, as shown in Figure 4d, minimized the polymer covering the filler due to the high shear stress that occurred during the extrusion process, thus maximizing the filler alignment compared to Figure 4e [9]. Moreover, in a classical converging die, the filler is induced to orientate longitudinally to the extrusion direction through the die thickness, while in a diverging die, an elongated deformation is created which will lead the filler to randomly orientate in the core of the die. Despite that, the diverging die encounters a major drawback compared to the converging die, as the fillers are unable to orientate in the desired direction due to the occurrence of shear deformations near the die wall [9,20].

**Figure 5:**
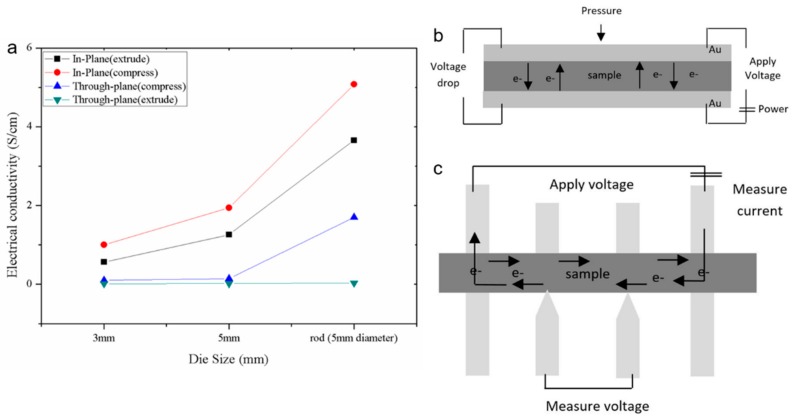
(**a**) Electrical conductivity of extruded and compress MCF/PP, schematic of (**b**) through-plane conductivity and (**c**) in-plane conductivity.

Figure 5 indicates that the MCFs produced using the rod-shaped die showed better electrical conductivity compared to those produced using other die shapes. However, the extruded composite produced higher electrical conductivity after undergoing the compression moulding process, as shown in Figure 5. Figure 5a shows the in-plane and through-plane electrical conductivity of the polymer composite when it is extruded and after it has been compressed. The schematic diagram of both in-plane and through-plane conductivity is also illustrated in Figure 5b,c. Theoretically, the in-plane conductivity allowed the electron to pass along the plate surface. Meanwhile, the through-plane allowed the electron to pass throughout the plate. The extruded MCF/PP produced using the rod-shaped die recorded the highest in-plane electrical conductivity (3.66 S/cm), while the ones produced using the sheet die with a thickness of 3 mm and 5 mm recorded an electrical conductivity of 0.56 and 1.26 S/cm, respectively. The higher electrical conductivity of the extruded composites produced by the rod-shaped die was due to the longer fiber size and higher orientation angles, which aid in producing an excellent electrical network formation, as illustrated in Figure 4c,f. However, the electrical conductivity of the extruded composite produced using the 5 mm thick sheet die was higher than that produced using the 3 mm thick sheet die. This was expected due to the high filler breakage at increased shear stress in the narrow dies, as shown in Figure 4a,b [14]. It is important to note that the volume fraction and fiber length certainly affect the viscosity of the composites at a high shear rate. However, the viscosity of the composites appeared to have been affected by the clustering of the fibers at a low shear stress, as observed in Figure 4b [33].

Meanwhile, the electrical conductivity of composite polymer improved after being hot pressed using the compression moulding process, as shown in Figure 5. The in-plane electrical conductivity of the rod die increased to 5.1 S/cm, while the 5 and 3 mm sheet dies increased to 2.0 and 1 S/cm, respectively. These results indicated that the electrical conductivity was improved after being compressed as the orientation angle remains as shown in Figure 4. Rod dies obtained the highest orientations angle of 84° compared to the 3 and 5 mm sheet die with 68° and 55°, respectively. Results exhibit that the orientation angle was maintained after the compression molding process while producing a compact structure. Meanwhile, Figure 4g,h show the existence of a void which deteriorates the electrical conductivity of the polymer composite. However, the void vanished as the compact structure was produced using the rod dies. Besides, the incomplete melt of polypropylene uncoated the filler, as shown in Figure 4g,h, thus deteriorating the formation of the conductive network. These results explain why the through-plane conductivity of the sheet dies remained unchanged compared to the rod die. As the aligned fiber tends to compress in the rod die, the contact between the fiber tends to increase. Hence, this causes an increase in electrical conductivity experience in the rod dies. In contrast, the electrical conductivity remains the same in the sheet dies due to the random orientate fiber, which cause no changes in fiber contact, as shown in Figure 4g,h. Different observations are shown in Figure 4i, where the fillers tend to align more in parallel to the extrusion direction after being compressed. Meanwhile, the through-plane electrical conductivity of the polymer composite increases drastically when using rod die compared to sheet dies. These results are explained by the synergistic effect of aligned fillers parallel to extrusion directions [14,41].

### 4.4. Mechanical Properties

The mechanical properties of the extruded composite materials, including density, flexural strength, and tensile strength, are reported in Table 2. The flexural strength and tensile strength of the extruded polymer composites seemed higher for those composites that were produced using the rod die with 25 and 1225 MPa, respectively, compared to those produced using the sheet die. This result was consistent with the increased electrical conductivity shown by the extruded composites produced using the rod die. Although the fillers were not fully coated with the polymer for the composites produced by the rod die, the strength within the filler and the polymer matrix were strong enough to resist being repelled by the filler, which explains why the mechanical properties and electrical conductivity of the composite produced using the rod die were higher. In addition, the polymer had already adhered to the filler in the produced composites due to the high aspect ratio of the filler [24,49]. The flexural strength results obtained in this study were higher compared to current studies using the epoxy as the polymer resin with the average of 60 MPa [29]. In a previous study using PEI-based carbon fibres, it was reported that an increase in the angle of orientation resulted in a drastic deterioration in the mechanical properties of the composite [50]. However, in the study using the extruded MCF/PP polymer composite, it was indicated that the tensile strength of the composite increased as the filler was aligned 90° to the extrusion direction, as shown in Table 2. This phenomenon occurred due to the fact that the twisted and aligned filler underwent the tensile breakage, whereby additional energy was required to break filler stitches [51]. However, the tensile modulus of the pure PP polymer was higher at 1.6 GPa, as reported by other researchers, compared to 1.2 GPa obtained in this study [52]. This is due to the fact that mechanical properties of composite materials tend to deteriorate as the filler breakage increases, as shown in Figure 4.

The hardness of the MCF/PP polymer composite is shown in Table 2. The extruded MCF/PP composite produced using the sheet die with a thickness of 5 mm showed the highest shore hardness (52.58D), while the extruded MCF/PP composite produced using the rod die showed the lowest shore hardness (43.5D). The increased shore hardness shown by the composites that were produced using the sheet dies was expected due to the improved dispersion and random orientation of the filler in the polymer matrix, which served to effectively harden the composites. Besides, the filler was coated with the polymer, as illustrated in Figure 4, which clearly explains the reason for the increased shore hardness of the composites produced using the sheet dies. However, the hardness of the polymer composite improves as the material is being compressed.

### 4.5. Effectiveness of Electrical Conductivity on Mechanical Properties

Recently, the development of composite materials with high electrical conductivity while maintaining the mechanical properties has been an issue in modern industries. Thus, this study applied an extrusion process which aligned the fiber 90° to the extrusion direction. This further improves the electrical conductivity as an excellent conductive pathway is developed. The extruded MCF/PP materials demonstrate the highest electrical conductivity of 5.1 S/cm rod dies after being compressed, as shown in Figure 5, due to an increase in compactions within the composite matrix [19]. Despite that, the rod die samples also improve the mechanical properties of composite materials in terms of flexural strength and tensile modulus with 25 and 1225 MPa, respectively, as shown in Table 2. This is evidence that improving the manufacturing process will aid in improving the electrical and mechanical properties of composite materials as the fiber tends to orientate accordingly during the extrusion process [53]. These findings are crucial as they produce an alternative in the manufacturing process which is cost and time effective, especially in fabricating a bipolar plate used in polymer electrolyte membrane fuel cells (PEMFC).

### 4.6. Electrical Conductivity Model

**Figure 6:**
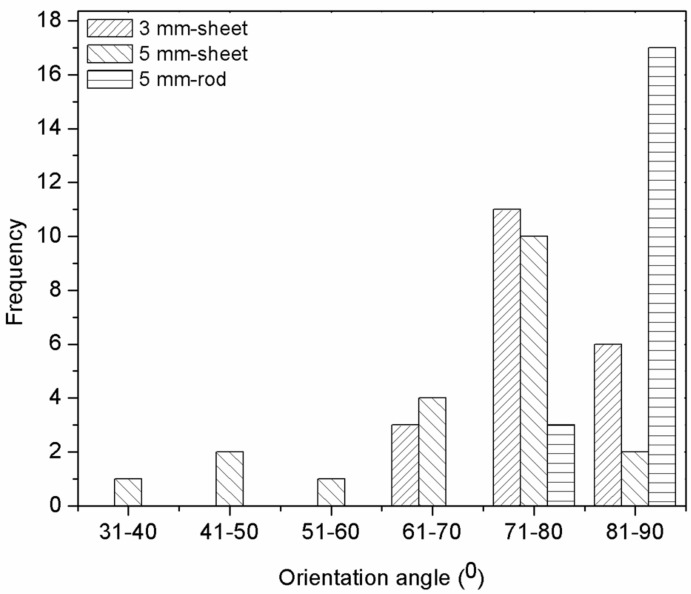
Histogram of filler orientation angle of extruded MCF/PP.

Figure 6 shows the filler orientation angle of the extruded polymer composite at different die sizes where the 5 mm diameter rod dies had the angle closest to the extrusion direction at 86°. Details of the filler orientation can be observed in Figure 6, where the histogram shows that the greater part of the filler was aligned at 90° to the extrusion direction, especially in the rod die. Meanwhile, the fillers in the other sheet dies were distributed and dispersed randomly as the filler counts were visible at an orientation angle of 90°. The results that were obtained were supported by the study by Hine in 1995, which reported that the converging dies are better able to align fibers in the extrusion direction compared to slit dies [16].

**Figure 7:**
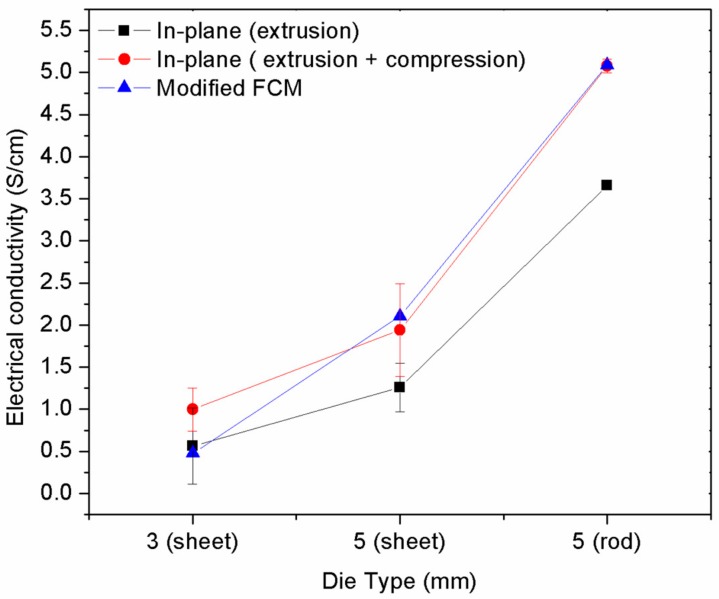
Electrical conductivity of extruded MCF/PP using the modified FCM model.

Based on the orientation angle measured from the experimental data, the semi-empirical model was used to predict the electrical conductivity of the MCF/PP polymer composite. The modified FCM model was applied as this model considers the orientation in the parameter [3]. In order to compare the model prediction with the commonly used semi-empirical model, the GEM model was also applied. The parameters used in the modified FCM model are listed in Table 3. The value of each parameter was identified based on the fiber used. Figure 7 shows the comparison of both the theoretical model and the experimental data of the modified FCM model where a good pattern was indicated between the experimental data and the theoretical model. The predicted electrical conductivity seemed to be lower than the experimental data obtained due to the limitation in defining the diameter of the filler contact variable, as it could not be measured directly from the image analysis. Therefore, the filler contact was assumed to be between 10^−9^ and 10^−10^ cm. Unlike the modified FCM, the GEM model predicted a value of 0.28 S/cm for the electrical conductivity of the overall die geometry. This was due to the limitation of the model, in that it could only take into account the filler composition and electrical conductivity [3].

## 5. Conclusions

The effects of the die configurations of polypropylene reinforced milled carbon fibers on the electrical conductivity and mechanical properties were studied using the extrusion technique as a pre-mixing process before being compressed using a compression moulding process. The electrical conductivity and mechanical properties of the MCF/PP polymer composite that was produced using a 5 mm diameter rod die were found to be higher compared to those produced using the sheet die with thicknesses of 3 mm and 5 mm. The tensile modulus and flexural strengths were 1225 MPa and 25.05 MPa, respectively, for the extruded MCF/PP produced using a rod die with a 5 mm diameter, and 945 and 13.93 MPa, respectively, for the extruded MCF/PP produced using the sheet die. The in-plane and through-plane electrical conductivities for the rod die increase after being compressed from 3.66 to 5.08 S/cm and 0.03 to 1.7 S/cm, respectively. The improved properties shown by the MCF/PP produced using the rod die were due to the fact that the greater part of the filler was aligned to the extrusion direction and tended to be more compact after being compressed. The compact filler also further improved the mechanical properties of the compressed polymer composite. In conclusion, the laminated rod dies produced a composite with better properties and performance compared to the composite produced using the sheet die.

## Figures and Tables

**Table 1:** Relative densities of different die types.

Type	Size	Density, *ρ*_s_ (g/cm^3^)	Relative Density (*ρ*_s_/*ρ*_TH_) (%)
Sheet (Thickness)	3 mm	1.194	96.06
	5 mm	1.2	96.54
Rod (∅)	5 mm	1.192	95.89

**Table 2:** Mechanical properties of the MCF/PP composite.

Type	Size	Hardness (Extrude)	Hardness (Compress)	Flexural Strength (MPa)	Tensile Modulus (MPa)
Sheet (Thickness)	3 mm	47.42	47.8	14.93	842
	5 mm	52.58	53.9	13.93	945
Rod (∅)	5 mm	43.5	50.6	25.05	1225

**Table 3:** Parameters of the modified FCM model.

Paraneters Symbol	Values
$\sigma_{m}$	10^−16^ S/cm
$\sigma_{f}$	100 S/cm
*d*	9 µm
*l*	300 µm
*φ*	0.7
*d_c_*	10^−9^ cm

## References

[1] Jimenez G.A., Jana S.C. (2007). Electrically conductive polymer nanocomposites of polymethylmethacrylate and carbon nanofibers prepared by chaotic mixing. Compos. Part A Appl. Sci. Manuf..

[2] Harun W.S.W., Kamariah M.S.I.N., Muhamad N., Ghani S.A.C., Ahmad F., Mohamed Z. (2017). A review of powder additive manufacturing processes for metallic biomaterials. Powder Technol..

[3] Zakaria M.Y., Sulong A.B., Sahari J., Suherman H. (2015). Effect of the addition of milled carbon fiber as a secondary filler on the electrical conductivity of graphite/epoxy composites for electrical conductive material. Compos. Part B Eng..

[4] Mejía A., García N., Guzmán J., Tiemblo P. (2014). Extrusion Processed Polymer Electrolytes based on Poly(ethylene oxide) and Modified Sepiolite Nanofibers: Effect of Composition and Filler Nature on Rheology and Conductivity. Electrochim. Acta.

[5] Nakayama Y., Takeda E., Shigeishi T., Tomiyama H., Kajiwara T. (2011). Melt-mixing by novel pitched-tip kneading disks in a co-rotating twin-screw extruder. Chem. Eng. Sci..

[6] Zhang Z.-X., Gao C., Xin Z.X., Kim J.K. (2012). Effects of extruder parameters and silica on physico-mechanical and foaming properties of PP/wood-fiber composites. Compos. Part B Eng..

[7] Kostic M., Reifschneider L. (2006). Design of extrusion dies. Encycl. Chem. Process..

[8] Samsudin M.S.F., Ishak Z.A.M., Jikan S.S., Ariff Z.M., Ariffin A. (2006). Effect of filler treatments on rheological behavior of calcium carbonate and talc-filled polypropylene hybrid composites. J. Appl. Polym. Sci..

[9] Fan Z., Advani S.G. (2005). Characterization of orientation state of carbon nanotubes in shear flow. Polymer.

[10] Tang W., Santare M.H., Advani S.G. (2003). Melt processing and mechanical property characterization of multi-walled carbon nanotube/high density polyethylene (MWNT/HDPE) composite films. Carbon N. Y..

[11] Kuriger R.J., Alam M.K., Anderson D.P., Jacobsen R.L. (2002). Processing and characterization of aligned vapor grown carbon fiber reinforced polypropylene. Compos. Part A Appl. Sci. Manuf..

[12] Wang Z., Fan X., Wang K., Deng H., Chen F., Fu Q. (2011). Fabrication of polypropylene/carbon nanotubes composites via a sequential process of (rotating solid-state mixing)-plus-(melt extrusion). Compos. Sci. Technol..

[13] Kim Y.A., Hayashi T., Endo M., Gotoh Y., Wada N., Seiyama J. (2006). Fabrication of aligned carbon nanotube-filled rubber composite. Scr. Mater..

[14] Taipalus R., Harmia T., Zhang M.Q., Friedrich K. (2001). The electrical conductivity of carbon-fibre-reinforced polypropylene/polyaniline complex-blends: Experimental characterisation and modelling. Compos. Sci. Technol..

[15] Ausias G., Jarrin J., Vincent M. (1996). Optimization of the tube-extrusion die for short-fiber-filled polymers. Compos. Sci. Technol..

[16] Hine P.J., Davidson N., Duckett R.A., Ward I.M. (1995). Measuring the fibre orientation and modelling the elastic properties of injection-moulded long-glass-fibre-reinforced nylon. Compos. Sci. Technol..

[17] Kakati B.K., Yamsani V.K., Dhathathreyan K.S., Sathiyamoorthy D., Verma A. (2009). The electrical conductivity of a composite bipolar plate for fuel cell applications. Carbon N. Y..

[18] Wang J., Geng C., Luo F., Liu Y., Wang K., Fu Q., He B. (2011). Shear induced fiber orientation, fiber breakage and matrix molecular orientation in long glass fiber reinforced polypropylene composites. Mater. Sci. Eng. A.

[19] Antunes R.A., de Oliveira M.C.L., Ett G., Ett V. (2011). Carbon materials in composite bipolar plates for polymer electrolyte membrane fuel cells: A review of the main challenges to improve electrical performance. J. Power Sources.

[20] Feller J.F., Linossier I., Grohens Y. (2002). Conductive polymer composites: Comparative study of poly(ester)-short carbon fibres and poly(epoxy)-short carbon fibres mechanical and electrical properties. Mater. Lett..

[21] Feller J.F., Chauvelon P., Linossier I., Glouannec P. (2003). Characterization of electrical and thermal properties of extruded tapes of thermoplastic conductive polymer composites (CPC). Polym. Test..

[22] Dweiri R., Sahari J. (2008). Microstructural image analysis and structure–electrical conductivity relationship of single- and multiple-filler conductive composites. Compos. Sci. Technol..

[23] Suherman H., Sahari J., Sulong A.B. (2013). Effect of small-sized conductive filler on the properties of an epoxy composite for a bipolar plate in a PEMFC. Ceram. Int..

[24] Alomayri T., Shaikh F.U.A., Low I.M. (2014). Effect of fabric orientation on mechanical properties of cotton fabric reinforced geopolymer composites. Mater. Des..

[25] Tungjitpornkull S., Sombatsompop N. (2009). Processing technique and fiber orientation angle affecting the mechanical properties of E-glass fiber reinforced wood/PVC composites. J. Mater. Process. Technol..

[26] Dweiri R., Sahari J. (2007). Electrical properties of carbon-based polypropylene composites for bipolar plates in polymer electrolyte membrane fuel cell (PEMFC). J. Power Sources.

[27] Liang J.Z., Peng W. (2009). Melt viscosity of PP and FEP/PP blends at low shear rates. Polym. Test..

[28] Planes E., Flandin L., Alberola N. (2012). Polymer Composites Bipolar Plates for PEMFCs. Energy Procedia.

[29] Mohd Radzuan N.A., Yusuf Zakaria M., Sulong A.B., Sahari J. (2017). The effect of milled carbon fibre filler on electrical conductivity in highly conductive polymer composites. Compos. Part B Eng..

[30] Mohd Radzuan N.A., Sulong A.B., Sahari J. (2017). A review of electrical conductivity models for conductive polymer composite. Int. J. Hydrogen Energy.

[31] Barton R.L., Keith J.M., King J.A. (2007). Development and modeling of electrically conductive carbon filled liquid crystal polymer composites for fuel cell bipolar plate applications. J. New Mater. Electrochem. Syst..

[32] Kakati B.K., Sathiyamoorthy D., Verma A. (2011). Semi-empirical modeling of electrical conductivity for composite bipolar plate with multiple reinforcements. Int. J. Hydrogen Energy.

[33] Köpplmayr T., Milosavljevic I., Aigner M., Hasslacher R., Plank B., Salaberger D., Miethlinger J. (2013). Influence of fiber orientation and length distribution on the rheological characterization of glass-fiber-filled polypropylene. Polym. Test..

[34] Keith J.M., King J.A., Barton R.L. (2006). Electrical conductivity modeling of carbon-filled liquid-crystalline polymer composites. J. Appl. Polym. Sci..

[35] Tavares L.B., Rocha R.G., Rosa D.S. (2017). An organic bioactive pro-oxidant behavior in thermal degradation kinetics of polypropylene films. Iran. Polym. J..

[36] Eberhardt C., Clarke A., Vincent M., Giroud T., Flouret S. (2001). Fibre-orientation measurements in short-glass-fibre composites—II: A quantitative error estimate of the 2D image analysis technique. Compos. Sci. Technol..

[37] Hobbie E.K., Wang H., Kim H., Lin-Gibson S., Grulke E.A. (2003). Orientation of carbon nanotubes in a sheared polymer melt. Phys. Fluids.

[38] Dweiri R. (2015). The Potential of Using Graphene Nanoplatelets for Electrically Conductive Compression-Molded Plates. JJMIE.

[39] Dweiri R., Sahari J. (2007). Computer simulation of electrical conductivity of graphite-based polypropylene composites based on digital image analysis. J. Mater. Sci..

[40] Radzuan N.A.M., Sulong A.B., Rao Somalu M. (2017). Electrical properties of extruded milled carbon fibre and polypropylene. J. Compos. Mater..

[41] Suherman H., Sulong A.B., Sahari J. (2013). Effect of the compression molding parameters on the in-plane and through-plane conductivity of carbon nanotubes/graphite/epoxy nanocomposites as bipolar plate material for a polymer electrolyte membrane fuel cell. Ceram. Int..

[42] Sulong A.B., Ramli M.I., Hau S.L., Sahari J., Muhamad N., Suherman H. (2013). Rheological and mechanical properties of carbon nanotube/Graphite/SS316L/polypropylene nanocomposite for a conductive polymer composite. Compos. Part B Eng..

[43] Liu S.-P., Hwang S.-S., Yeh J.-M., Hung C.-C. (2011). Mechanical properties of polyamide-6/montmorillonite nanocomposites—Prepared by the twin-screw extruder mixed technique. Int. Commun. Heat Mass Transf..

[44] Adloo A., Sadeghi M., Masoomi M., Pazhooh H.N. (2016). High performance polymeric bipolar plate based on polypropylene/graphite/graphene/nano-carbon black composites for PEM fuel cells. Renew. Energy.

[45] Mustafa A., Muhammed Ali S.A., Abdalla A.M., Somalu M.R., Muchtar A. (2017). Effect of sintering temperature on the microstructure and ionic conductivity of Ce0.8Sm0.1Ba0.1O2-δ electrolyte. Process. Appl. Ceram..

[46] Muhammed Ali S.A., Anwar M., Abdalla A.M., Somalu M.R., Muchtar A. (2017). Ce0.80Sm0.10Ba0.05Er0.05O2-δ multi-doped ceria electrolyte for intermediate temperature solid oxide fuel cells. Ceram. Int..

[47] Muhammed Ali S.A., Anwar M., Somalu M.R., Muchtar A. (2017). Enhancement of the interfacial polarization resistance of La0.6Sr0.4Co0.2Fe0.8O3-δ cathode by microwave-assisted combustion method. Ceram. Int..

[48] Pötschke P., Bhattacharyya A.R., Janke A. (2004). Melt mixing of polycarbonate with multiwalled carbon nanotubes: Microscopic studies on the state of dispersion. Eur. Polym. J..

[49] Taherian R., Hadianfard M.J., Golikand A.N. (2013). Manufacture of a polymer-based carbon nanocomposite as bipolar plate of proton exchange membrane fuel cells. Mater. Des..

[50] Dhakate S.R., Sharma S., Borah M., Mathur R.B., Dhami T.L. (2008). Development and characterization of expanded graphite-based nanocomposite as bipolar plate for polymer electrolyte membrane fuel cells (PEMFCs). Energy Fuels.

[51] Solaimurugan S., Velmurugan R. (2008). Influence of in-plane fibre orientation on mode I interlaminar fracture toughness of stitched glass/polyester composites. Compos. Sci. Technol..

[52] Zhou Y., Rangari V., Mahfuz H., Jeelani S., Mallick P.K. (2005). Experimental study on thermal and mechanical behavior of polypropylene, talc/polypropylene and polypropylene/clay nanocomposites. Mater. Sci. Eng. A.

[53] Huang G., Han Y., Guo X., Qiu D., Wang L., Lu W., Zhang D. (2017). Effects of extrusion ratio on microstructural evolution and mechanical behavior of in situ synthesized Ti-6Al-4V composites. Mater. Sci. Eng. A.

